# Evaluating Ecohydrological Theories of Woody Root Distribution in the Kalahari

**DOI:** 10.1371/journal.pone.0033996

**Published:** 2012-03-28

**Authors:** Abinash Bhattachan, Mokganedi Tatlhego, Kebonye Dintwe, Frances O'Donnell, Kelly K. Caylor, Gregory S. Okin, Danielle O. Perrot, Susan Ringrose, Paolo D'Odorico

**Affiliations:** 1 Department of Environmental Sciences, University of Virginia, Charlottesville, Virginia, United States of America; 2 Okavango Research Institute, University of Botswana, Maun, Botswana; 3 Department of Geography, University of California Los Angeles, Los Angeles, California, United States of America; 4 Department of Forestry and Range Resources, Gaborone, Botswana; 5 Department of Civil and Environmental Engineering, Princeton University, Princeton, New Jersey, United States of America; 6 Department of Geography, University of Colorado, Boulder, Colorado, United States of America; 7 School of Architecture, Civil and Environmental Engineering, École Polytechnique Fédérale De Lausanne, Lausanne, Switzerland; Lakehead University, Canada

## Abstract

The contribution of savannas to global carbon storage is poorly understood, in part due to lack of knowledge of the amount of belowground biomass. In these ecosystems, the coexistence of woody and herbaceous life forms is often explained on the basis of belowground interactions among roots. However, the distribution of root biomass in savannas has seldom been investigated, and the dependence of root biomass on rainfall regime remains unclear, particularly for woody plants. Here we investigate patterns of belowground woody biomass along a rainfall gradient in the Kalahari of southern Africa, a region with consistent sandy soils. We test the hypotheses that (1) the root depth increases with mean annual precipitation (root optimality and plant hydrotropism hypothesis), and (2) the root-to-shoot ratio increases with decreasing mean annual rainfall (functional equilibrium hypothesis). Both hypotheses have been previously assessed for herbaceous vegetation using global root data sets. Our data do not support these hypotheses for the case of woody plants in savannas. We find that in the Kalahari, the root profiles of woody plants do not become deeper with increasing mean annual precipitation, whereas the root-to-shoot ratios decrease along a gradient of increasing aridity.

## Introduction

Savannas are mixed plant communities with tree and grass species coexisting in the same landscape. They cover about 20% of the global land surface, including approximately one-half of Africa and Australia, 45% of South America and 10% of India and Southeast Asia [1], [2], [3]. Savannas are home to a large portion of the human population, and provide important ecosystem services such as rangelands for livestock grazing [2] and carbon storage [4], [5]. Investigating root distribution in savanna vegetation and associated soil carbon pools is an important step towards the assessment of the global carbon budget [6].

Research in savanna ecology has long-recognized four major determinants of ecosystem structure, namely: fire, herbivory, water, and nutrient availability [1], [3], [7], [8], [9], [10]. The structure of savanna vegetation is strongly governed by the spatiotemporal distribution of these four factors. Vegetation structure may, in turn, play a major role in the distribution of abiotic resources such as energy, water and nutrients [2], [11], [12]. For example, tree canopies redistribute rain water [13] and shade the ground, reducing the rates of soil evaporation and maintaining higher moisture levels in the subcanopy soils [2], [11]. Similarly, the presence of tree roots and higher soil organic matter content enhance soil infiltration capacity beneath trees [14], [15]. As a result, the dynamics of soil moisture are strongly affected by the distribution of tree canopies and their accompanying root systems [11], [16], [17].

Processes governing vegetation composition and structure in savannas have been, for the most part, inferred from the study of patterns of aboveground plant biomass. Canopy cover, tree spacing, and the amount of woody biomass in relation to herbaceous biomass have been often considered the descriptors of vegetation structure in these ecosystems (e.g., [18], [19], [20]). However, the dynamics of savanna vegetation are strongly dependent on belowground processes such as competition for water and nutrients [21], [22], [23]. Root distribution affects plants' ability to compete for soil resources [2], [16], [24], ecosystem carbon storage [13], [14], [25] and water redistribution within the soil profile [26], [27], [28]. Some classic theories of tree-grass coexistence and plant geographic distribution are based on specific assumptions on root structure and function (e.g., [29], [30]). For example, it has long been assumed (e.g., [29]) that tree-grass coexistence is due to niche separation between the woody and herbaceous life forms that exploit different soil layers with minimal overlap between their root zones (e.g., [31]). However, the assumption that woody plants grow most of their roots in deeper soil layers while herbaceous vegetation has roots only in the shallow soils has been repeatedly challenged by a number of studies [21], [24], [32], [33], [34]. Therefore new advances in savanna ecology require a better understanding of patterns of root distribution. Studies on rooting depth and root profiles are also essential for regional and global-scale assessments of belowground carbon storage and climate modeling, as they define the thickness of the soil layer exploited by plants for water and nutrient uptake (e.g., [35], [36]).

Two major issues need to be addressed to improve our current understanding of root structure and patterns of belowground biomass in savannas. First, the niche separation hypothesis is based on the assumption that savanna trees are deeply rooted. However, the rooting depth of woody plants in savannas remains poorly investigated. In fact, it is not clear how the rooting depth varies with different conditions of water availability. Theoretical studies have shown that optimal root profiles (i.e., the profiles that maximize plant transpiration while minimizing water stress) become deeper in wetter climates [37], [38], [39], [40], [41], [42]. However, field observations along aridity gradients have shown a good agreement with this theory only in the case of herbaceous vegetation, while no significant relationship has been found between the rooting depth of woody vegetation and precipitation [21]. A possible explanation could be that, while in water-limited ecosystems roots are expected to become deeper with increasing mean annual precipitation, in more mesic environments root profiles are determined also by nutrient limitations. Schenk [43] listed a number of other reasons why there is an advantage for roots to be shallow and noted that roots “tend to be as shallow as possible and as deep as needed to fulfill evapotranspirational demands”.

Second, the assessment of carbon storage in savannas requires a better understanding of the relation between above and belowground biomass and its dependence on the rainfall regime. Root systems are often referred to as “the hidden half” (e.g., [44]) and carbon budget studies sometime assume live belowground biomass to be similar in magnitude to the above ground biomass [45]. However, the relation between root and shoot biomass appears to be more complex [46]. It has been argued that plants have a way of regulating the growth of their above- and belowground biomass [47] maintaining a “functional equilibrium” [47], [48], [49]. According to this equilibrium theory, limitations in the availability of aboveground resources (for example, light) induce an increase in aboveground plant biomass (e.g., leaves), whereas root growth is stimulated by limitations in belowground resources. The “functional equilibrium theory” would suggest that the root-to-shoot ratio (R∶S, the ratio of belowground to aboveground biomass) should increase from mesic to xeric environments, due to the decrease in soil moisture levels and decrease in vegetation density and competition for light. Patterns of belowground primary production are consistent with the “functional equilibrium hypothesis” in the case of herbaceous vegetation in tropical savannas [50]. However, it remains to be seen whether this hypothesis can explain patterns of above- and belowground woody biomass in savannas.

Although global scale reviews (e.g., [4], [21], [51], [52]) are in agreement with ecological theories on the relationship between root structure and water availability (e.g., [30], [37], [47]), they tend to mix studies made on different soil textures and presumably using different sampling methods. To avoid these issues, we investigate root depths and R∶S ratios at sites in the Kalahari, where a relatively homogeneous sandy soil exists along a latitudinal rainfall gradient [53], [54]. This unique observational setting allows us to examine the “optimal rooting depth” (e.g., [21], [37], [38], [42], [52]) and the “functional equilibrium” [47] theories of root structure across a large gradient of climate independent of major changes in soil type. More specifically, we will use data from a major root sampling effort to study how the rooting depth of woody plants and the ratio between belowground and aboveground biomass vary along the Kalahari's rainfall gradient.

## Methods

### Study sites

The Kalahari sand sheet is one of the largest continuous sand deposits on Earth, stretching for several thousand kilometers across a rainfall gradient, with no substantial variability in the soil physical properties [54]. Known as the Kalahari Transect (KT), this region is an ideal natural laboratory for ecohydrologic global change studies [55], [56] in that the effects of changing hydrologic conditions can be investigated without the confounding effects of different soil types.

**Figure 1:**
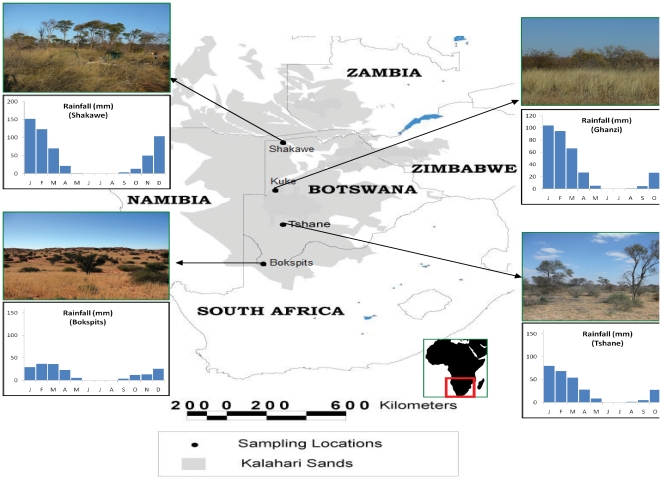
Geographic location of the study region and of the research sites.

This study involves four field sites located within the Botswana portion of the KT (Fig. 1), along a south-north rainfall gradient ranging between 180 and about 550 mm/yr with an average storm depth of 10 mm d^−1^ [57]. More details on the seasonality of precipitation are provided in Figure 1. All sites exhibit savanna vegetation found on a very thick (>100 m) homogeneous sandy substrate with consistent physical and geochemical properties. The grain size analysis of soil collected at depths 0–10 cm, 10–30 cm, 30–70 cm, and 70–120 cm shows that soils are consistently sandy (>90% sand) throughout the top 1.2 m profile and across the entire transect (Table 1). Given the geomorphic context (i.e., the Kalahari sand sheet) there is no reason to believe that soil texture differences would play a role in root distribution. Water table depth ranges from about 25 m at the wettest site (Shakawe) to about 100 m deep at the driest (Bokspits). Vegetation composition and structure vary across the study region from an open fine-leaf shrub savanna in the south, through a bush savanna, to a mixture of bush and broad leaf woodland savanna in the northwestern part of Botswana [58].

**Table 1:** Grain size analysis of the soil samples was conducted using a particle size analyzer (LS 13 320, Beckman Coulter®).

Site	Depth (m)	% clay	% silt	% sand
**Shakawe**	0.1	0.6	1	98.4
	0.3	0.7	1.7	97.6
	0.7	0.6	0.7	98.7
	1.2	0.5	0.7	98.8
**Ghanzi**	0.1	3	1	96.0
**Tshane**	0.1	0.9	4.1	95.0
	0.3	1	5.9	93.1
	0.7	1	6.8	92.2
	1.2	1.1	8.2	90.7
**Bokspits**	0.1	0.8	3.1	96.1
	0.3	0.7	2.1	97.2
	0.7	0.7	2.6	96.7
	1.2	0.6	2.7	96.7

Soil samples were collected from depths 0–0.1 m, 0.1–0.3 m, 0.3–0.7 m, and 0.7–120 m at all sites except for the Kuke/Ghanzi area, where we have used only soils from the top 10 cm.

Individual study site characteristics (i.e. location, rainfall regime and dominant woody and herbaceous vegetation) are summarized in Table 2. Mean annual precipitation (MAP) values used in this study were calculated using meteorological records (Botswana Bureau of Meteorology, 1971–2000) from Shakawe, Ghanzi, Tshane, and Bokspits. All necessary permits were obtained (from the Ministry of Environment, Wildlife and Tourism of the Republic of Botswana) to perform the described field studies at these sites.

**Table 2:** Coordinates, mean annual precipitation (MAP), and plant community composition of the study sites.

Site	Coordinates	MAP (mm)	Main Woody Species	Main Grass Species
**Shakawe**	18°21′51″S, 21°50′31″E	539±170 (209.4)	Terminalia sericea, Ochna pulchra, Pterocarpus angolensis, Burkea africana	Aristida meridionales, Eragrostis spp, Schmidtia pappophoroides
**Kuke**	20°58′36″S, 22°28′48″E	439±157 (162.2)	Terminalia serecia, Lonchocapus nelssi, Acacia erubescence, Acacia fleckii	Eragrostis lehmanniana, Schmidtia pappophoroides, Anthphora pubescenes
**Tshane**	24°01′01″S,21°52′08″E	358±133 (156.5)	Lycium species, Acacia mellifera, Acacia luderitzii	Aristida congesta, Eragrostis lehmanniana, Eragrostis pallensis
**Bokspits**	26°53′39″S 20°41′54″E	177±107 (55.2)	Rhigozum_trichotomum Acacia mellifera, Acacia erioloba, Boscia albitrunca	Stipagrostis amabilis, Stipagrostis uniplumis, Schtmidtia kalahariensis

Information on precipitation includes mean annual precipitation (MAP) ± the standard deviation of annual precipitation and (in parentheses) the minimum annual precipitation recorded in 1971–2006. Because rainfall data from Kuke do not exist, the values reported are from Ghanzi.

### Field scale surveys of above- and below-ground biomass

The survey of aboveground and belowground biomass took place during the dry season over the course of three field campaigns (June–August) in 2008 (in Shakawe), 2009 (in Bokspits and Tshane), and 2010 (in Kuke and Tshane). At each of the four sites, three 20 m×20 m plots were established in randomly selected locations within 1000 m of each other. In each plot, the spatial location of each tree and shrub was mapped and plant height, tree stem coordinates, basal diameter, and canopy size were measured. The aboveground tree and shrub biomass was then harvested and weighed. An estimate of aboveground wet biomass in g m^−2^ was calculated by dividing the total aboveground biomass by the plot area (400 m^2^).

### Root sampling

A variety of methods are commonly used to investigate root structure and function, including microsatellite markers [59], tracer uptake [22], [60], ground penetrating radar [61], minirhizotron cameras [62], coring, and excavations using shovels or air pressure systems (e.g., [44], [63]). While non-destructive methods (e.g., tracer-based methods) are used to determine the zones of influence [22], [60] of plant roots and the function of fine roots [64], they are not effective at quantifying woody root structure and biomass in that only functional (i.e., active) roots contribute to tracer uptake. Root excavation is the most direct approach to determine root biomass because it samples both active and inactive roots, and allows for direct root mass measurement. Therefore, excavation methods were utilized to quantify belowground biomass. Each plot was partitioned into a square grid of 20×20 square subplots of 1 m×1 m area. Twenty of these 1 m^2^ subplots were randomly selected in each plot (i.e., a total of 60 plots per site). Each of these 1 m^2^ subplots were dug to a depth of 110 cm, with a 30 cm-thick surface layer and four subsequent 20 cm-thick layers.

During the excavation of these 1 m^2^ soil pits, all woody roots in each soil layer were harvested, cleaned of soil, and weighed; length and diameter were measured with a caliper. Roots finer than 2 mm in diameter were grouped and then weighed together for each soil layer. The same procedure was repeated in all three plots at each of the four study sites for a total of 240 soil pits. In 2009, a subset of both root and branch pieces of various lengths and widths were collected at the Tshane site. These samples were oven-dried and reweighed to determine the ratio between dry and wet biomass as explained in section 2.4.

### Data Analyses

A relation between dry and wet biomass (*k* = wet mass/dry mass) was determined from a subset of roots (n = 50), stems (n = 75) and branch (n = 102) samples collected in the field and weighted both before and after oven drying for 24 hours at 60°C. This subset of samples was used to perform an analysis of covariance (ANCOVA) on the ratio of fresh mass to dry mass of each sample using tissue type (i.e., above ground or below ground biomass) and species as factors and size of the wet sample as a covariate. It was found that, while for above ground biomass the *k* ratio does not significant vary across the three species here considered (i.e., *k* =  Boschia albutrunca, Terminalia sericea, and Acacia mellifera), significant differences in the *k* ratios of below ground biomass existed among the same species. However, because the sampling protocol used for belowground biomass consisted of excavating a number of soil pits, and collecting and measuring the root samples in each pit, we were unable to determine to what species each sample belonged to. For this reason, we used the same *k* ratio for all above ground biomass samples and another one for all the belowground samples, without accounting for differences among species. The analysis of covariance was then repeated using tissue type (i.e., above ground or below ground biomass) as a factor and size of the wet sample as a covariate. The results (Table 3) show that *k* is significantly different for aboveground (*k* = 1.64) and belowground (*k* = 1.87) biomass. For aboveground biomass there was a significant interaction (*p* = 0.259) between *k* and the size of the sample (expressed in terms of wet biomass); however, the slope of the interaction between branch and wet mass is close to zero (see Table 3) and the effect of this interaction on the estimate of aboveground biomass is overall negligible. Conversely for belowground biomass the interaction was not significant (*p* = 0.0253). The above analysis was then used to convert wet biomass to dry biomass with two different values of *k* for roots and stems or branches.

**Table 3:** Results of the analysis of covariance performed on the wet to dry biomass ratio, *k*, using tissue type (i.e., above ground or below ground biomass) as a factor and size of the wet sample as a covariate.

Variable	Estimate	Std. Error	t - value	p - value
*k* (aboveground)	1.64	0.0264	61.9	1.33E-138
*k* (roots)	1.87	0.0372	50.2	2.80E-120
*k*(aboveground): wet mass	−0.000387	0.000342	−1.13	0.259
*k*(roots): wet mass	0.00128	0.000570	2.25	0.0253

Plot-level averages of aboveground biomass, belowground biomass, and root-to-shoot ratio were tested for normality using the Jarque-Bera test [65] at the 5% significance level. To determine how rainfall influences allocation, we calculated the correlation coefficient of MAP and each of these variables with the plot as the unit of replication.

Root biomass data were expressed in terms of root density, r (z), (i.e., dry root mass per unit volume of soil) and analyzed to assess changes with depth and across sites. For each of the 20×20 plots we fit an exponential distribution, r(z) =  a exp[−bz], to the data where a and b are two parameters obtained by fitting the cumulative distribution, 
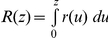
, to the data. Following Schenk and Jackson [52], we expressed root depth in terms of the portions, Z_50_ and Z_95_, of the soil profile, which comprise 50% and 95% of the total root biomass, respectively. The 50% and 95% percentiles were derived from the fitted equations, while the total belowground biomass for the whole soil profile (in g m^−2^) was calculated by extrapolation as R(∞) = a/b. The same analysis was then repeated for the vertical profiles of average linear root density, r_L_(z), defined as the average root length per unit volume of soil. Based on the linear root density, the depths, Z_L,50_ and Z_L,95_ - containing 50% and 95% of the total root length, respectively - were calculated.

## Results

**Figure 2:**
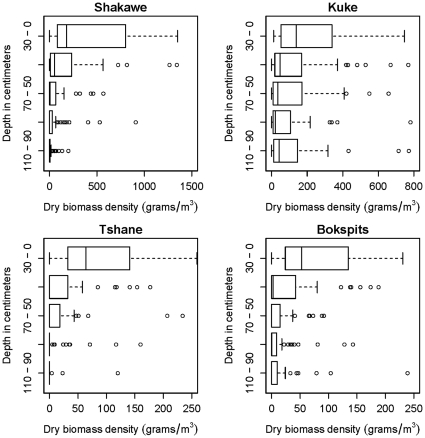
Dry biomass density of roots in the four sites across the Kalahari aridity gradient. The error bars indicate the minimum and maximum data values, unless outliers are present (shown as circles). The black line indicates the median, while the box boundaries are the lower and upper quartiles. Based on a set of 60 soil profiles sampled at each site.

**Figure 3:**
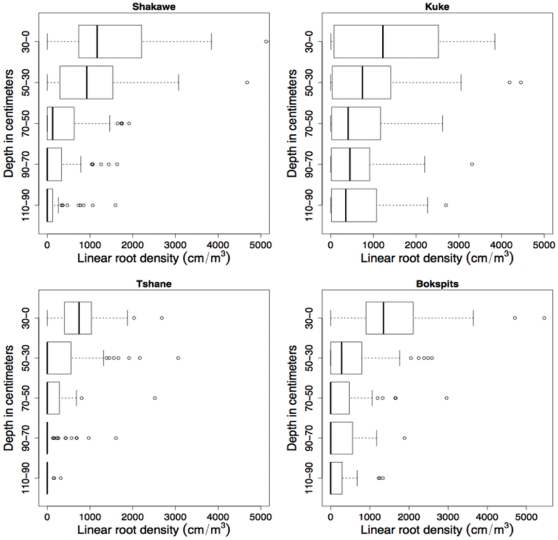
Linear root density (average root lengths per volume) across the Kalahari rainfall gradient in Botswana. Kuke site exhibits the highest length/volume readings compared to the other sites. The error bars indicate the minimum and maximum data values, unless outliers are present (shown as circles). The black line indicates the median, while the box boundaries are the lower and upper quartiles. Based on a set of 60 soil profiles sampled at each site.

**Figure 4:**
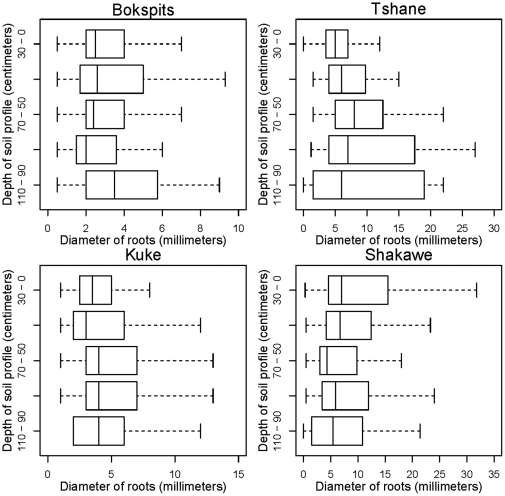
Root diameter distribution along the soil profile across the Kalahari's rainfall gradient. The error bars indicate the minimum and maximum data values, unless outliers are present (shown as circles). The black line indicates the median, while the box boundaries are the lower and upper quartiles. Based on a set of 60 soil profiles sampled at each site.

Root biomass decreases with depth at all sites except Kuke, which exhibits a relatively uniform distribution of root biomass in the top 1.10 m (Fig. 2). At all sites, an exponential distribution provides a good fit of the field data (R^2^ = 0.99 for each of these four sites, see Table 4). The 95% confidence bounds reported in Table 4 show that at Kuke the *b* parameter is not significantly different from zero, suggesting that at this site the root profile is uniform. Moreover, these bounds show that at all sites the within-site variability was smaller than the variability of *a* and *b* among sites. An indicator of root distribution, such as the linear root density (Fig. 3), exhibits a well-defined exponential decrease with depth at all sites (R^2^>0.98 at all sites, see Table 4), while root diameters do not exhibit a well-defined dependence on depth (Fig. 4).

**Table 4:** Parameters, *a* and *b*, and associated statistics from the fitting of an exponential distribution *r*(*z*) = *a* e^−*bz*^ to the average root profile in the vertical (*z*) direction.

Site	a (g m^−3^)	b (m^−1^)	R^2^	RMSE (g m^−3^)
	*a_L_* (cm m^−3^)	*b_L_* (m^−1^)	*R^2^*	*RMSE* (cm m^−3^)
	[95% confidence bounds]	[95% confidence bounds]		
**Shakawe**	5250 [4833.0, 5668.0]	4.874 [4.425, 5.323]	0.9995	18.8
	*7595* [*4911*, *10280*]	*1.367* [*0.3384*, *2.396*]	*0.9834*	*229.68*
**Kuke**	540.4 [404.0, 676.7]	0.2413 [−0.334, 0.817]	0.9928	33.5
	*5555* [*5121*, *5988*]	*0.6726* [*0.4773*, *0.868*]	*0.9993*	*48.52*
**Tshane**	330.0 [271.8, 388.1]	1.636 [1.093, 2.180]	0.9959	8.3
	*3946* [*3223*, *4670*]	*2.37* [*1.71*, *3.03*]	*0.9957*	*42.54*
**Bokspits**	257.7 [204.8, 310.5]	2.775 [1.974, 3.576]	0.9948	5.0
	*7299* [*6709*, *7889*]	*2.298* [*2.011*, *2.584*]	*0.9991*	*35.62*

The parameters *a_L_*, *b_L_*, and associated statistics from the fitting of an exponential distribution *r_L_*(*z*) = *a_L_* e*^−b^_L_^z^* to the average root density (figure 3) are shown in italic fonts. The distribution is calculated as an average of three plots (20 soil pits per plot) at five depths.

Overall, tree roots in the Kalahari are relatively shallow with Z_50_ depths (i.e., 50% of root biomass is shallower than Z_50_) ranging between 0.14 and 0.43 m (Table 5), while the Z_95_ depths (i.e., 95% of the roots are shallower than Z_95_) range between 0.61 and 1.88 m. The only exception to this is the Kuke site, where the root profile in the top 1.10 m was relatively uniform. Therefore, at this site the estimation of root depth by fitting an exponential function provided values of Z_50_ and Z_95_ that were much larger than the 1.10 m depth explored in our excavations. Thus, these values of Z_50_ and Z_95_ are not reported in Table 5. Linear root density profiles (e.g., Z_L,50_) exhibit relatively shallow distributions at all sites.. The rooting depths reported in Table 5, however, do not show any clear relationship with MAP since the deepest root systems were found in areas in the middle of the transect (Kuke and Tshane) rather than at either end. Similarly, neither the interannual variability nor the minimum values of precipitation (see Table 2) could explain root depth variability among sites.

**Table 5:** Z_50_ and Z_95_ root depths calculated assuming an exponential profile of root biomass.

Site	Z_50_ (m)	Z_95_ (m)	Root Biomass in Top 1.10 m (g/m^2^)	Extrapolated Total Root Biomass, R(∞) (g/m^2^)	Above-ground Biomass (g/m^2^)	Basal Area (m^2^ ha^−1^)	R∶S (top 1.1 m)	Z_L,50_ (m)	Z_L_,_95_ (m)
Shakawe	0.14	0.61	1070.76±676.9	1078.03	438.65±272.46	3.6	3.10±2.91	0.26	1.16
Kuke	n/a	n/a	536.16±100.34	n/a	842.08±236.53	6.4	0.64±0.08	0.90	3.89
Tshane	0.43	1.88	163.75±40.44	201.71	513.37±68.20	2.1	0.30±0.03	0.31	1.32
Bokspits	0.25	1.08	91.89±28.75	92.86	556.50±116.76	1.9	0.17±0.03	0.28	1.25

Measured dry belowground biomass (top 1.1 m), extrapolated total root biomass (i.e., R(∞), see Methods section), dry above ground biomass, basal area, root-to-shoot ratio (R∶S, based on dry root biomass in the top 1.10 m), and depths, Z_L,50_ and Z_L,95_, of soil column containing 50% and 95% of the total root length, respectively (assuming exponential profile of linear root density). Biomass variability among plots expressed as ± standard deviation calculated for a set of 3 plot replicates at each site.

**Figure 5:**
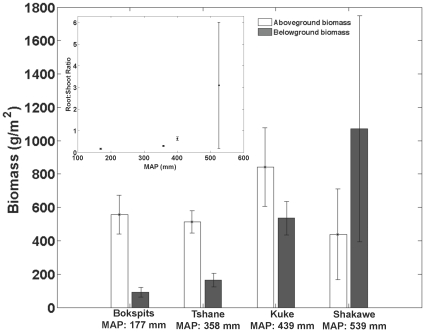
Measured average woody plant biomass (above and belowground) per unit area (top 1.1 m) across the Kalahari transect aridity gradient. Inset: ratio of below to above ground biomass. The error bars represent ± standard deviation calculated for a set of 3 plot replicates at each site.

The Jarque-Bera test indicated that above- and belowground biomass and R∶S ratio were not normally distributed, but the log transforms of these variables are. Belowground biomass decreases with decreasing MAP (Table 5) with relatively large variations among sample plots, particularly at the wetter sites (Fig. 5). The correlation coefficient of MAP and log-transformed belowground biomass was 0.90 (p<0.01) for a set of 3 plot replicates at each site. The same relationship can be also found in the extrapolated values of total belowground biomass, R(∞). Nonetheless, the plots used in this study did not show any clear dependence of aboveground woody biomass on MAP (Fig. 5). There was no significant correlation between log-transformed aboveground biomass and MAP (p = 0.66) for a set of 3 plot replicates at each site. However, in fire-prone areas above ground biomass is difficult to estimate because of its dependence on the time since last disturbance. The ratio of below- to aboveground biomass has a clear positive relationship with rainfall, with a correlation between MAP and log-transformed R∶S ratio of 0.89 (p<0.001). Shakawe exhibited the highest R∶S ratio and Bokspits the lowest (Fig. 5-inset), indicating that woody plants at the more mesic (northern) sites allocate more of their biomass to roots relative to woody plants in the dryer (southern) sites.

## Discussion

We found that there is a positive relationship between belowground biomass and mean annual rainfall, which is consistent with previous studies in other systems [5], [52]. However, because there is no clear relationship between above ground biomass and annual rainfall at our sites, the R∶S ratio increases with rainfall (Fig. 5), which is inconsistent with the results by Schulze et al. [5].

According to the functional equilibrium hypothesis of plant growth [48] plants should allocate more carbon to belowground biomass in areas with stronger limitations in belowground resources (e.g., soil moisture), while they should allocate more carbon to above ground biomass in areas that are more limited by above ground resources (e.g., light). Thus, this hypothesis predicts decreasing R∶S with increasing MAP. For studies on biomass allocation between below- and aboveground biomass in grasses and in potted plants some authors [48], [50], [66], [67] have shown that the R∶S ratios increase with aridity (i.e., decrease with increasing MAP). Our data for woody species in the Kalahari directly contradict the predictions of the functional equilibrium hypothesis. The inconsistency between our results and those from studies of potted plants and herbaceous vegetation suggests that different vegetation growth forms respond differently to climatic and environmental drivers (e.g. [5], [21], [33]).

In recent years, theories of root distribution [37], [38], [39], [40], [41], [68] have provided a framework to determine “optimal root profiles”, i.e., the vertical root distributions that optimize the use of soil moisture by water limited vegetation for given climate and soil properties. According to these theories, optimal root profiles in semiarid environments have the following characteristics: 1) the root density profile decreases exponentially with depth (e.g., [37]), and 2) root depth (e.g., Z_50_ and Z_95_ or Z_L,50_ and Z_L,95_) increases with MAP due to the fact that rainwater infiltrates deeper into the soil column in mesic environments [11], [37], [38], [39] and plants grow more roots where there are more resources to take up, a phenomenon known as “hydrotropism” (e.g., [47], [69]).

Our data show that, in the Kalahari, the linear root density of woody species – an indicator of roots' ability to take up water and other soil resources (e.g., [35]) - decreases exponentially with depth at all sites (Fig. 3), but values of Z_L,50_ and Z_L,95_ calculated from these fits do not increase with increasing MAP. Indeed the lowest values of Z_L,50_ and Z_L,95_ occur at Shakawe, the wettest site. The same is observed with Z_50_ and Z_95_ calculated from average root mass profiles, though in this case there is one site (Kuke) with a root mass profile that does not exponentially decrease with depth (Fig. 2c) and for which Z_50_ and Z_95_ values cannot be calculated. Thus, our data do not support the predictions of optimality theories of root distribution.

Using data from different biomes and across a wide precipitation range, Schenk and Jackson [21] found that for grasses and forbs, the rooting depth exhibits a strong positive relationship with MAP. However, this relationship with MAP was less clear for trees and shrubs [21]. This study shows that woody vegetation does not exhibit an increase in rooting depth with MAP.

Overall, our data show that woody vegetation in the Kalahari has relatively shallow roots, despite the sandy texture of Kalahari soils, which would be expected to favor plants with deep roots because of the deeper and faster infiltration typical of soils with coarse texture [30]. The vegetation we sampled has 50% of its root biomass in the top 0.14 to 0.43 m of the soil column (and 95% in the top 0.61 or 1.88 m), except for the Kuke site, which exhibits deeper roots (Table 5).

**Figure 6:**
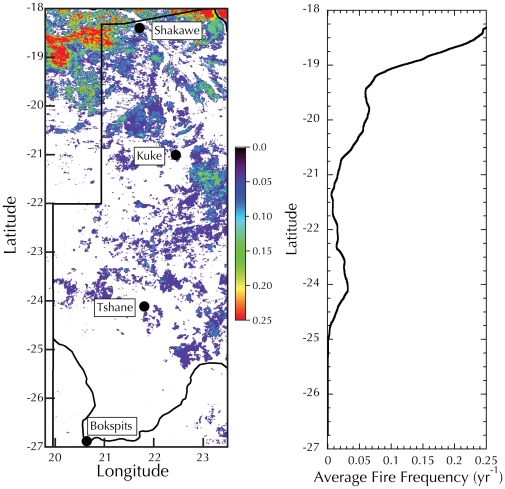
Left: Fire frequency from 2000 to 2011 calculated from the MODIS Burned Area Product (MCD45) [**72**] in yr^−1^. White areas experienced no fires during this period. Right: Average fire frequency (in yr^−1^) calculated along a longitudinal transect (21.3°) using a moving box of approximately 100×100 km.

Given the failure of existing theories to explain this new and unique dataset, alternative explanations of root distribution must be explored. We hypothesize that our data could emerge from differing strategies of water and carbon utilization and storage along the Kalahari Transect. In the Kalahari, as in savannas worldwide, woody vegetation must contend with ongoing threats of fire and drought. In the northern portion of the transect, rainfall is higher, and consequently, the incidence of fire during the dry season is also higher (see Fig. 6 and e.g., [70], [71]). In the southern portion of the transect precipitation is lower, so there is insufficient biomass to sustain significant fires (Fig. 6). Thus, different strategies are appropriate at different points along the rainfall gradient. We hypothesize that in the more mesic portion of the transect a strategy that stores carbon is better suited to the climatic and fire conditions. In this strategy, plants invest in significant belowground root systems to provide ample storage for water and carbon. Although the large root system brings with it significant metabolic costs, the ability for storage that it entails means that loss of the aboveground structure in a fire can be compensated for by quick growth from the belowground stores. Due to the additional cost of respiration required by this large belowground storage system, the plant may be in carbon deficit in some years (especially during droughts), but will still have excess metabolic carbon in most years. In contrast, the lack of fire and the more frequent drought occurrence in the dry portion of the transect favors a second strategy. In this strategy, the root system is not used for carbon and water storage, but is almost exclusively used for foraging of soil resources, mainly water. Because an extensive root system cannot be sustained in these water-limited environments, the plants have a lower R∶S ratio. Large belowground biomass is not required under this strategy because no storage to be used for re-growth of aboveground biomass is required where fire frequency is low. In this strategy, a small root system can harvest ample water to support the organism, as long as it is also shallow and spatially extensive, and does not provide a large metabolic burden, especially in areas where the risk of drought, and therefore carbon deficit, is high.

This hypothesis of life-history strategies explains several important features of our data, including why the R∶S ratio increases with increasing MAP (due to the need for additional storage as the risk of fire increases), and why rooting depth is shallow in the dry portion of the transect (so roots can easily access soil moisture from small precipitation events), while not requiring that rooting depth be deeper in wetter areas.
